# Physiological Responses and Metabonomics Analysis of Male and Female *Sargassum thunbergii* Macroalgae Exposed to Ultraviolet-B Stress

**DOI:** 10.3389/fpls.2022.778602

**Published:** 2022-04-04

**Authors:** Yan Sun, Qian Liu, Shuai Shang, Jun Chen, Peiyao Lu, Yu Zang, Xuexi Tang

**Affiliations:** ^1^College of Marine Life Sciences, Ocean University of China, Qingdao, China; ^2^Key Laboratory of Marine Eco-Environmental Science and Technology, First Institute of Oceanography, Ministry of Natural Resources, Qingdao, China; ^3^College of Biological and Environmental Engineering, Binzhou University, Binzhou, China; ^4^Laboratory for Marine Ecology and Environmental Science, Qingdao National Laboratory for Marine Science and Technology, Qingdao, China

**Keywords:** metabonomics, biomass allocation, dioecious, ultraviolet-B, *Sargassum thunbergii*

## Abstract

Ultraviolet-B (UV-B) radiation is a major environmental stress that suppresses or activates defense responses in organisms. UV-B radiation affecting growth and development in intertidal species have been researched for a long time, but a series of unknown knowledge remain in the male and female macroalgae comparison. To compare the different responses of male and female *Sargassum thunbergii* macroalgae under UV-B radiation, PSII photochemical efficiency determination, metabolomic analysis, and main carbon-based metabolites (including soluble sugar, total amino acid, and lipid) content measuring have been performed in our experiments. Results showed that males have significantly superiority performance in the chlorophyll fluorescence parameters of *F*_v_/*F*_m_, Y(II), and Y(NO) either low or high UV-B radiation treatments. Metabolomics analysis revealed that carbon and nitrogen metabolism pathways in male and female *S. thunbergii* were significant components responding to enhanced UV-B radiation. Based on measuring, female *S. thunbergii* lipid content expressed higher than males without any stimulation. Additionally, under low UV-B radiation stimulation, females total amino acid content shown significantly higher than control group and their lipid content also significantly higher than males. Under high UV-B radiation, males soluble sugar, total amino acid, and lipid content significantly varied from females, which meant that enhancing UV-B stress might altered mainly carbon-based metabolites flowing directions. The present study elucidated the potential role of enhanced UV-B radiation in regulating macroalgae physiological responses, metabolites changing, and reflecting differences between male and female *S. thunbergii*, contributing to understanding of brown-macroalgae diecious adopting mechanisms in defending intertidal UV-B stresses.

## Introduction

The effects of ultraviolet-B (UV-B) radiation on algae have been studied extensively (Holzinger and Lütz, 2006; Karsten and Holzinger, 2014; Holzinger et al., 2018); UV-B radiation inhibits survival, growth, membrane permeability, pigment composition, assembly of phycobiliprotein, carbon sequestration, and nitrogen assimilation (Donkor and Häder, 1991; Sinha et al., 1996). Previous research focused on the effects on growth and development of algae of UV-B radiation (Grobe and Murphy, 1997), biomass productivity (Renger et al., 1986; Khotimchenko and Yakovleva, 2005; Zeeshan and Prasad, 2009), photosynthesis/photoinhibition (Bischof et al., 2000a; Yakovleva and Titlyanov, 2001; Edgardo et al., 2013), photosynthetic pigments (Döhler and Buchmann, 1995; Döhler and Lohmann, 1995; Heo and Jeon, 2009), reactive oxygen accumulation (Mallick and Mohn, 2000), antioxidant system (Lee and Shiu, 2009), UV-absorbing compounds content (Huovinen et al., 2010), etc. Because of sea and land interactions, large scale growth of *Sargassum thunbergii* may involve diverse adaptive mechanisms and evolutionary information. As tides change, there are periodic changes in exposure to direct sunlight. Hence, compared to other algae in water, these intertidal plants such as *S. thunbergii* are more susceptible to the effects of UV-B. These algae evolved mechanisms to resist UV-B, include antioxidant protection mechanisms (Rijstenbil, 2002; Shiu and Lee, 2005), damage repair mechanisms (Häder and Sinha, 2005), and radiation-resistant material compensation mechanisms (Bischof et al., 2000b; Kräbs et al., 2002).

With the development of technology, metabolomics technology is no longer limited to traditional targeted metabolite chemical composition analysis. Instead, it is now applied to the quantitative and qualitative determination of metabolites from the perspective of systems biology and has been widely used in plant metabolism variance analysis. Hence, metabolomics analysis has become a technology for detecting changes in complex organisms (Khakimov et al., 2014) and provides a large amount of metabolic pathways information. Comparing to the traditionally biochemical method, genomics, and proteomics analysis methods, the metabolomics analysis can be carried out more quickly (Putri et al., 2013) and directly responds to the metabolites contents regulation as well as causing minimal damage to the experimental subjects (Ivanisevic et al., 2013). This emerging technology could provide us the global views of metabolites changing for comparison *S. thunbergii* males and females’ differential responses under UV-B radiation.

Dioecious plants play essential roles in maintaining terrestrial ecosystem stability and biodiversity conservation. Plants adapt under longterm natural selective pressure, gradually adapting to the environment; such adaptations include male and female heterosexual forms, male and female homoecious forms, and others (Hesse and Pannell, 2011). Sex-related responses to stresses were tested in dioecious plants to various stresses (Alvarez et al., 2013; Randriamanana et al., 2015; Chen et al., 2016). Dioecious plants showed significant gender bias in response to environmental stress such as cold damage, UV-B radiation, disease, salinity tolerance, and flooding; male plants often showed better tolerance and defense mechanisms than female plants. Previous studies also have shown that females of *Morus alba*, *Populus cathayana*, *Hippophae rhamnoides* (Li et al., 2004), and *Aciphylla glacialis* are more responsive than males, suffer greater negative effects under UV-B radiation stress. Compared with female plants, the basal diameter and leaf nitrogen content of male plants were significantly increased, and the chlorophyll content, UV-B absorption material, leaf area, and dry matter accumulation were significantly decreased. Male plants had higher photosynthetic rate and adaptability, and had more effective antioxidant system and higher anthocyanin content, so as to alleviate the adverse effects of UV-B radiation greater resistance to UV-B radiation (Pickering, 2000; Li et al., 2004; Xu et al., 2010; Chen et al., 2016). Most previous studies discussed different sexual dimorphism, physiological activities, and environmental tolerance in dioecious higher plants in response to stresses. Little attention has been paid to dioecious species of macroalgae that are important for marine ecosystem stability. Dioecious macroalgae are not only an important part of coastal ecosystem, but also an important dominant species in many algal ecosystems.

The macroalgae *S. thunbergii* is found in intertidal and shallow sublittoral zones of China, Japan, and Korea (Liu et al., 2016). It is one of the most dominant dioecious seaweeds in intertidal zones, characterized as transition zones between marine and terrestrial environments. Intertidal zones are among the most environmentally stressed regions on earth. Compared to other species, *S. thunbergii* is more exposed to the effects of UV-B radiation that induce light stress and aggravate conditions of thermal and desiccation stress (Chu et al., 2012a,b). Studying the adaptation mechanism of *S. thunbergii* to UV-B radiation stress is of great significance to protecting and restoring *S. thunbergii* resources and the construction of algae farms, it also fills the gap in understanding the molecular mechanism of the response of male and female macroalgae to environmental changes. However, relatively few studies have explored to dioecious macroalgae differences comparison and metabolomics technology could capture the precise physiological states of corresponding organisms. Potentially, there were might specific responding mechanisms or metabolic activities to UV-B radiation between different genders, which also closely related to their evolutionary process and environmental adaptation. So we chose *S. thunbergii* as a model to characterize regulation of physiology and their photosynthetic activities were measured to reflect the UV-tolerant differences between males and females. We also grasped the globally metabolic profiles changing under UV-B radiation, which can help us investigate gender respond differences and regulated mechanisms under UV-B stresses especially for addressing seaweed physiology questions.

## Materials and Methods

### Collection and Culture Conditions

Fertile *S. thunbergii* were collected on July 25, 2019, in the intertidal zone of Tai Ping Jiao (36°02′58.3″N, 120°21′31.9″E), Qing Dao Shandong province, China. For genders distinguishing, *S. thunbergii* matured reproductive receptacles have been observed before the experiments starting. On the one hand, the length of males matured reproductive receptacle (more than 1.5 cm) was significantly longer than female *S. thunbergii* (normally less than 0.9 cm). Additionally, during the reproductive periods, about 300–400 eggs adhered in female reproductive receptacles and their shapes were irregular oval observing by the microscope (Olympus, Tokyo, Japan), which could help us easier to distinguished *S. thunbergii* genders. Selected thalli (about 35–45 cm) were healthy and yellowish-brown in appearance with intact and firm receptacles with no apparent shedding. Specimens were transported to the laboratory within 1 h after collection, carefully scrubbed with a soft bristle brush to remove sand and small herbivores, and then rinsed with sterilized seawater several times.

We chose specimens of the same sizes and growth states from the available material and placed them in a 420 L glass tank (120 cm × 70 cm × 50 cm) filled with filtered seawater that was continuously aerated. The cultured conditions in tanks shown as follows: seawater taken from macroalgal grown places maintained with 18 ± 0.5°C at the 150 μmol m^−2^ s^−1^ and 14 L: 10 D (light: dark cycle) photoperiod photosynthetically active radiation (PAR). The light sources provided by the full-spectrum light-emitting diode (LED) lamp (AT1-Pro, Netlea, Guangdong province, China). And the characteristics of cultured seawater were approached to 31 ± 1 salinity, 8.0 ± 0.2 pH values replaced it every 2 days.

### Stress Treatments

For the UV treatments, UV radiation and PAR were provided by a combination of fluorescent lamps: UV-B was provided by a Philips Ultraviolet-B TL 40 W/12 RS (spectral range from 280 to 315 nm) with a primary output at 312 nm; PAR was provided by an OSRAM L 36W/32 Lumilux deluxe warm white and Radium NL 36W/26 Universal white light source. The UV-B radiation system adopted the method of artificially increasing UV-B radiation under laboratory conditions: (150 μmol photons·m^−2^ s^−1^) + UV-B (0 W·m^−2^ s^−1^). The UV-B radiation intensities controlled by the 0.12 mm bore-diameter cellulose acetate films and determined by the 297 UV irradiator (Beijing Normal University), respectively. In order to set the different illumination conditions, the distance of the set of lamps was adjusted trying to avoid major changes in the spectral quality of the emission. Three groups were set in our experiments including control group (only PAR), low UV-B radiation (PAR + 1 W·m^−2^ s^−1^ UV-B), and high UV-B radiation (PAR + 2.5 W·m^−2^ s^−1^ UV-B) treatment, which were continued 8 h radiation in experimental every day.

### Chlorophyll Fluorescence Parameters Determination

The pulse amplitude-modulated (PAM) fluorometer (Imaging-PAM fluorometer, Walz, Effeltrich, Germany) was used to measure the PSII chlorophyll fluorescence parameters and then male and female *S. thunbergii* macroalgae’ photosynthetic system activities under UV-B radiation enhancement were analyzed. At the experiments beginning, distinguishing and labeling genders *S. thunbergii* were selected and directly proceed PAM analysis after different intensities UV-B radiation exposing 8 h. Dioecious *S. thunbergii* bionts were dark-acclimated for 30 min prior to measurement. The *F*_v_/*F*_m_, Y(II), Y(NPQ), and Y(NO) at day 1, 3, and 5 were, respectively, measured and calculated (0.8 s 4,000 μmol photons·m^−2^·s^−1^ saturation pulse treatment, 30 s interval, and duration 600 s recording) with fluorescence induction curves (ICs; Kramer et al., 2004).

### Metabolic Analysis and Data Processing

In samples preparation, control groups, low UV-B radiation group, and high UV-B radiation group contained 5, respectively, male and female macroalgae. The exposing time was 8 h everyday and the samples were collected after 3 day treatment. The samples were thawed at 4°C and mixed with 1 ml of cold methanol/acetonitrile/water (2:2:1, *v*/*v*/*v*). The homogenates were sonicated at low temperature (30 min/once, twice). The mixture was centrifuged for 20 min (14,000 *g*, 4°C). The supernatants were dried in a vacuum centrifuge. For LC-MS (liquid chromatography-mass spectrometry) analysis, the samples were re-dissolved in 100 μl acetonitrile/water (1:1, *v*/*v*).

For HILIC (hydrophilic interaction liquid chromatography) separation, samples were analyzed using a 2.1 mm × 100 mm ACQUIY UPLC BEH 1.7 μm column (Waters, Ireland). In electrospray ionization (ESI) positive and negative modes, the mobile phase contained A = 25 mM ammonium acetate and 25 mM ammonium hydroxide in water, and B = acetonitrile. The gradient was 85% B for 1 min and was linearly reduced to 65% in 11 min, then reduced to 40% in 0.1 min and kept for 4 min, then increased to 85% in 0.1 min, with a 5 min re-equilibration period.

For reversed phase liquid chromatography (RPLC) separation, a 2.1 mm × 100 mm ACQUIY UPLC HSS T3 1.8 μm column (Waters) was used. In ESI positive mode, the mobile phase contained A = water with 0.1% formic acid and B = acetonitrile with 0.1% formic acid; in ESI negative mode, the mobile phase contained A = 0.5 mM ammonium fluoride in water and B = acetonitrile. The gradient was 1% B for 1.5 min and was linearly increased to 99% for 11.5 min and maintained for 3.5 min. Then, it was reduced to 1% in 0.1 min, and a 3.4 min re-equilibration period was employed. The gradients were at a flow rate of 0.3 ml min^−1^, and the column temperatures were kept constant at 25°C. A 2 μl aliquot of each sample was injected. The stability of the experimental system was assessed by comparing the total ion chromatogram of the quality control samples.

The raw mass spectroscopy data (wiff.scan files) were converted to MzXML files using Proteo Wizard MS Convert before importing into freely available XCMS software. For peak-picking, the following parameters were used as: centWave *m*/*z* = 25 ppm, peak width = c (10, 60), and prefilter = c (10, 100). For peak grouping, bw = 5, mzwid = 0.025, and minfrac = 0.5 were used. Only the variables having more than 50% of the non-zero measurement values in at least one group were kept in the extracted ion features. Compound identification of metabolites using MS/MS spectra with an in-house database was established using available authentic standards.

After normalization to total peak intensity, the processed data were uploaded before importing into SIMCA-P (version 14.1, Umetrics, Umea, Sweden). They were subjected to multivariate data analysis, including Pareto-scaled principal component analysis and orthogonal partial least-squares discriminant analysis (OPLS-DA). Sevenfold cross-validation and response permutation testing were used to evaluate the robustness of the model. The variable importance in the projection (VIP) value of each variable in the OPLS-DA model was calculated to indicate its contribution to the classification. Metabolites with the VIP value >1 were further applied to Student’s *t*-test at the univariate level to measure the significance of each metabolite. Values of *p* less than 0.05 were considered statistically significant. Metabolite difference multiples were calculated using the Student’s *t*-test according to the expression levels in the two comparison groups. As a commonly used exploratory data analysis method, hierarchical clustering analysis was carried out, which aims to group and classify changed metabolites based on similarity. In the clustering analysis process, the clustering algorithm classifies the two dimensions of samples and variables (usually refers to the quantitative information of proteins/metabolites/genes) and the results of chromatographic clustering were represented by tree-type heat map, for example, the red and blue represented up- and downregulated, respectively. In our results, Z-score was used to standardize the peak intensity, and MeV software (TigerLogic Co., Irvine, United States) was used for hierarchical clustering analysis of samples based on Euclidean distance algorithm to reflect the change trend of metabolites. Using KEGG (Kyoto Encyclopedia of Genes and Genomes)^1^ pathway as unit, Fisher’s exact test was used to analyze and calculate the significance level of metabolite enrichment in each pathway to determine the metabolic and signal transduction pathways that were significantly affected. The heat map was made through the APT-Biocloud.^2^

### Physiological and Biochemical Indexes Measurement

A plant soluble sugar content test kit (Nanjing Jiancheng Bioengineering Institute, China) was used to determine sugar content. The amino acid content was tested using a Total Amino Acid Content Assay Kit (Nanjing Jiancheng Bioengineering Institute, China). Lipid content was measured using a triglyceride content determination kit (Nanjing Jiancheng Bioengineering Institute, China). Succinate dehydrogenase (SDH) was determined using kits (Solarbio, China), according to the manufacturer’s instructions.

The SDH activities, amino acid, and lipid content were measured using BCA protein assay kits (Nanjing Jiancheng Bioengineering Institute, China) and the fresh weight (FW) was used to determine the *S. thunbergii* soluble sugar content; 0.1 g of frozen macroalgae was homogenized with 1 ml phosphate-buffered (0.1 mol, pH 7.0). The mixture was centrifuged at 4°C. Then, the four indicators in the supernatant were detected using the kits mentioned above.

### Statistical Analysis

SPSS 25.0 statistical software was used for physiological and biochemical experiments data analysis. Data were checked for normality and homogeneity of variances, and log-transformed to correct deviations from these assumptions when needed. The experimental data were analyzed by one-way ANOVA between different genders and Tukey’s multiple comparison at a probability of 0.05 was also used. Statistical significance was set at *p* < 0.05.

## Results

### The Effects of UV-B Radiation on Dioecious *Sargassum thunbergii* PSII Photosynthetic Parameters

**Figure 1:**
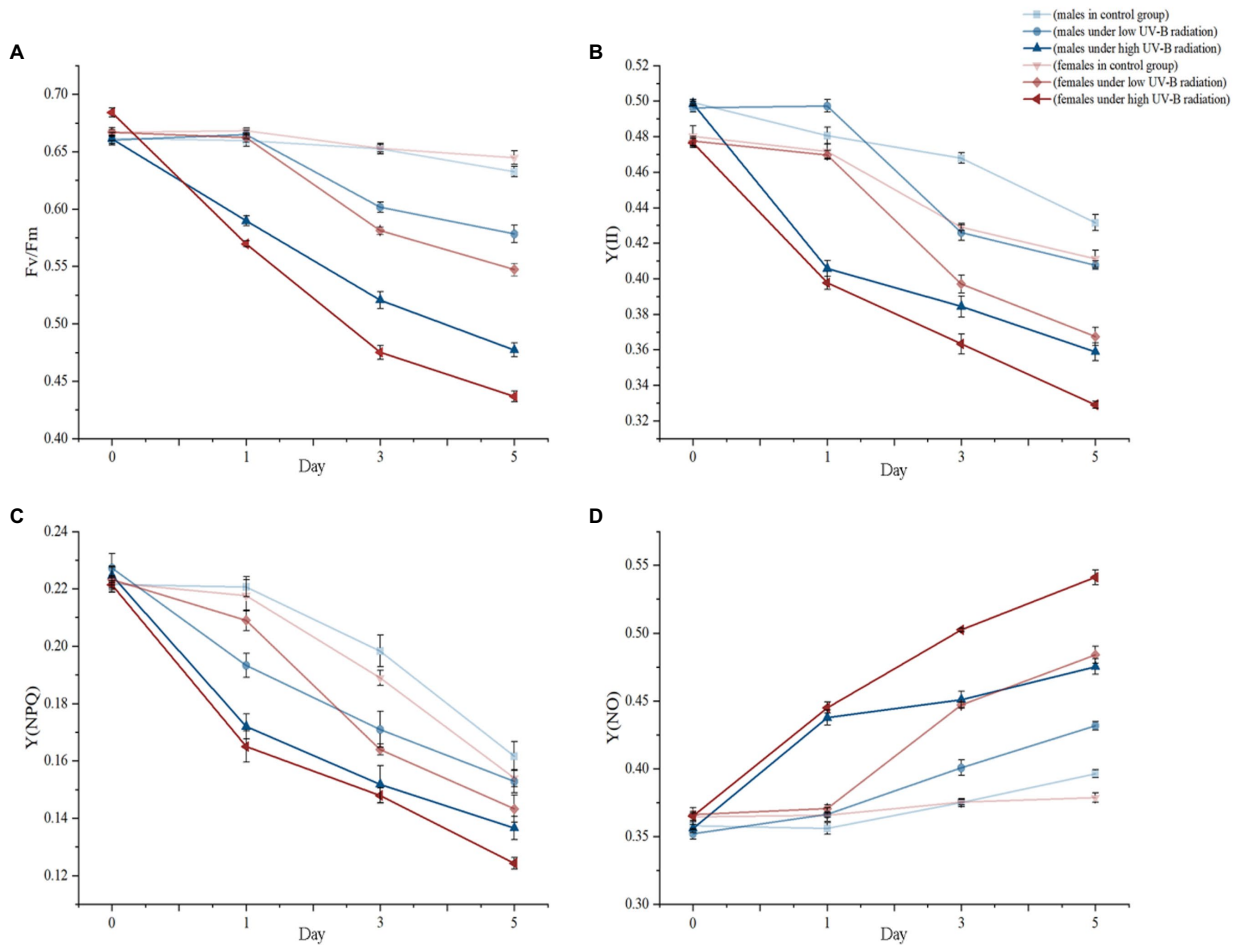
The PSII parameters including **(A)** *F*_v_/*F*_m_, **(B)** Y(II), **(C)** Y(NPQ), and **(D)** Y(NO) changes of dioecious *Sargassum thunbergii* in different treatments during the 5 day experiments. The values represent mean *n* ± SD (*n* = 3).

The PSII chlorophyll fluorescence parameters of male and female macroalgae showed obvious gender differences responding to enhanced UV-B radiation (Figure 1). At the beginning of treatments, different intensities UV-B radiation had not caused significantly different performances in the values of *F*_v_/*F*_m_, Y(II), Y(NPQ), and Y(NO) between males and females at day 1. However, after 3 days exposing, low and high UV-B radiation have significantly influenced three parameters including *F*_v_/*F*_m_, Y(II), and Y(NO) between males and females (*p* < 0.05). Comparing with the control group, low UV-B radiation caused significant 9.52% (*p* < 0.05) and 12.24% (*p* < 0.05) decreasing in males and females *F*_v_/*F*_m_ values, respectively. Analogously, higher UV-B radiation also significantly decreased males 11.70% and female 16.59% *F*_v_/*F*_m_ values. These similar tendencies also appeared decreasing in Y(II), Y(NPQ) versus to increasing in Y(NO). By dioecious *S. thunbergii* comparing, only Y(NPQ) has not shown significant differences under low or high UV-B radiation. After 5 days, stressed treatments, except for Y(NPQ), dioecious variation has been expanded consistently in low and high UV-B radiation according to day 3 values in *F*_v_/*F*_m_, Y(II) and Y(NO) and the quantitative values were shown in Supplementary Table S1.

### Metabolomics Results

#### Differentially Expressed Metabolites in Male and Female Macroalgae Under Enhanced UV-B Radiation

The strength and retention time of the various chromatographic peaks for the quality control (QC) samples overlapped, suggesting that the variation caused by instrument error was slight. The total numbers of peaks picked by XCMS for the positive and negative ion modes were 9,635 and 8,246, respectively.

**Figure 2:**
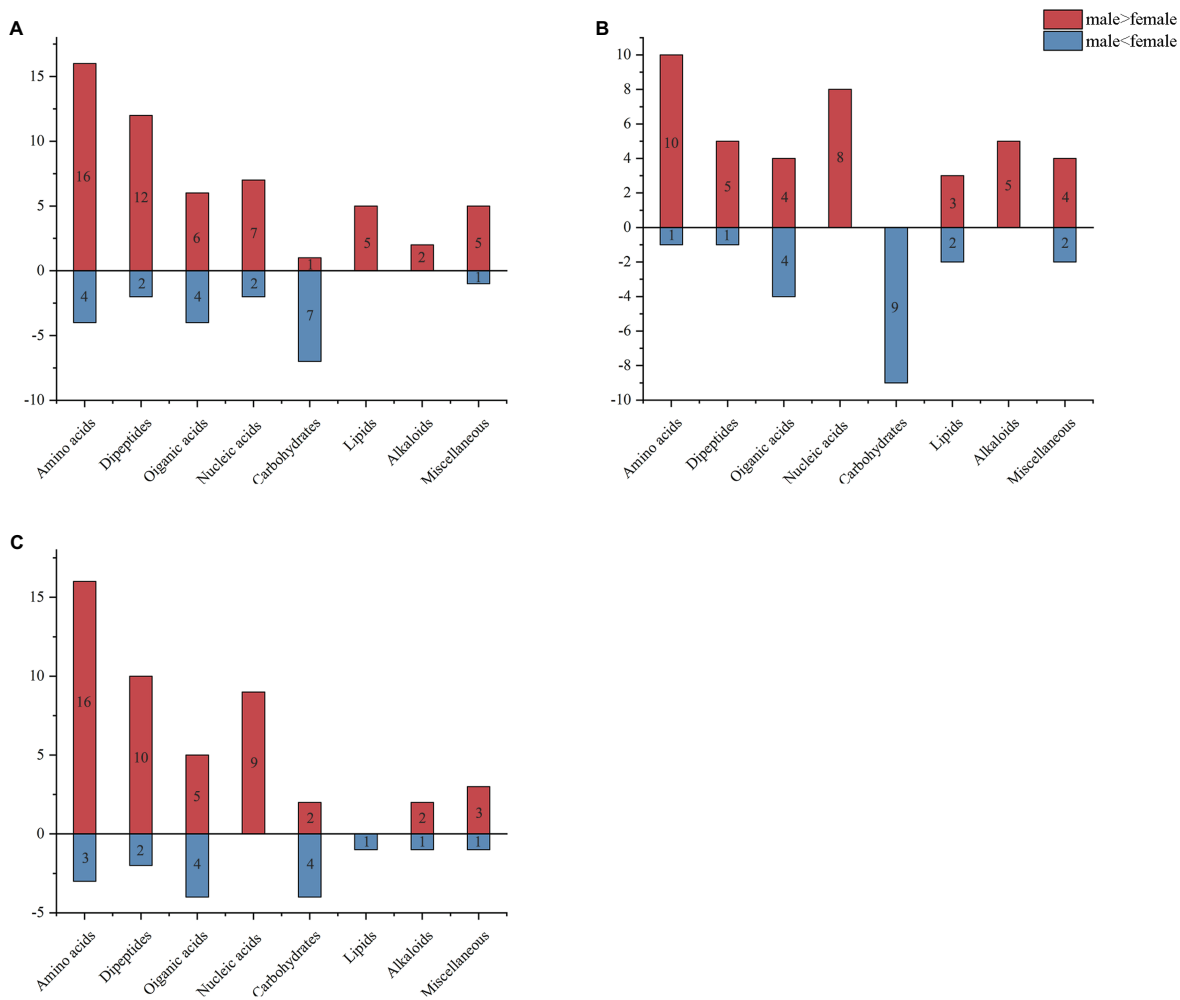
Metabolite analysis of male and female *Sargassum thunbergii* macroalgae under different UV-B radiation intensities. Male and female *Sargassum thunbergii* in **(A)** control group; **(B)** under low UV-B radiation conditions; and **(C)** under high UV-B radiation conditions. Red rectangle represents metabolite content in males significantly higher than females. Blue rectangle represents metabolite content in females significantly higher than males.

**Figure 3:**
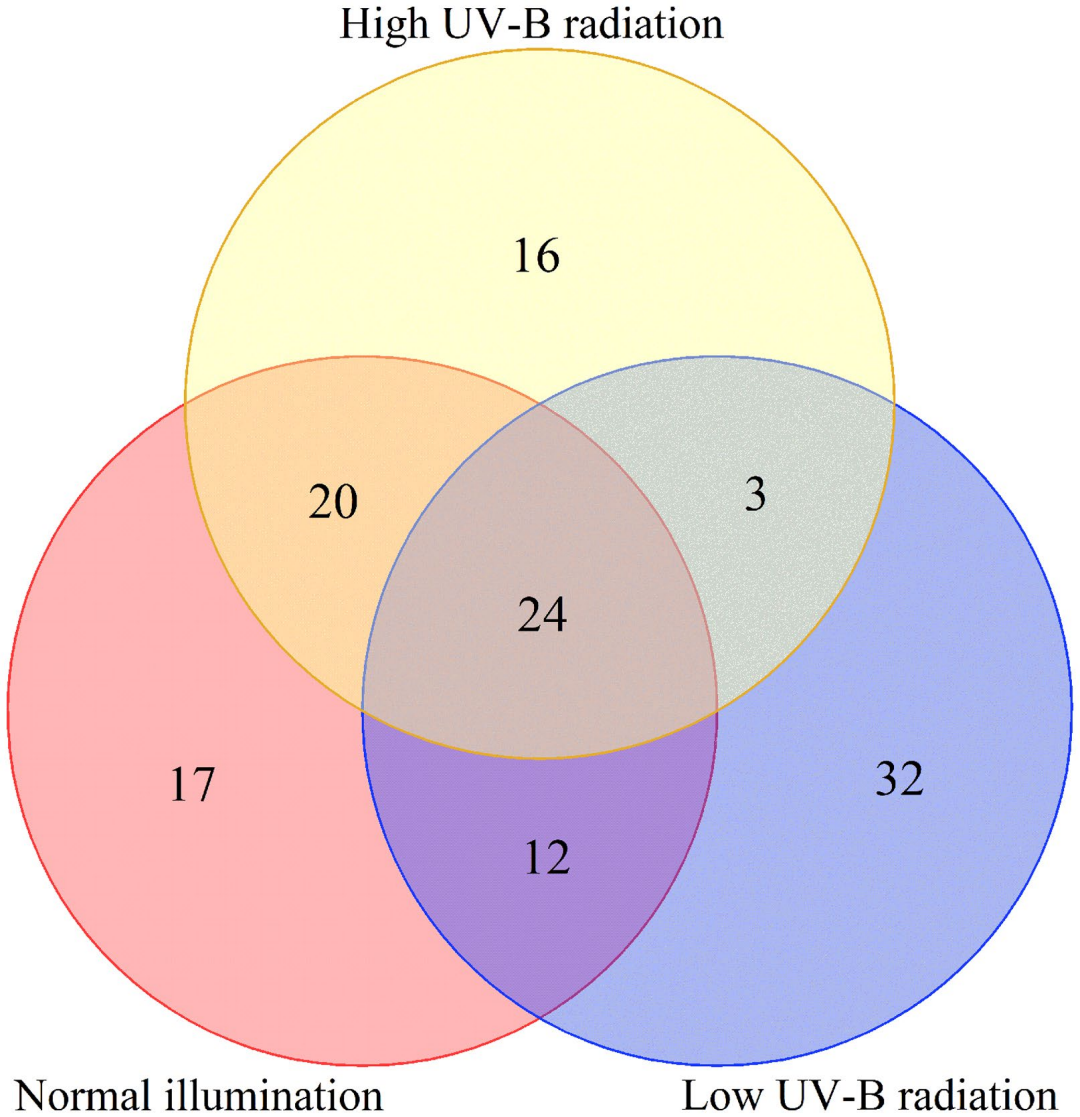
Venn diagram of differential metabolites under different UV-B radiation intensity.

**Figure 4:**
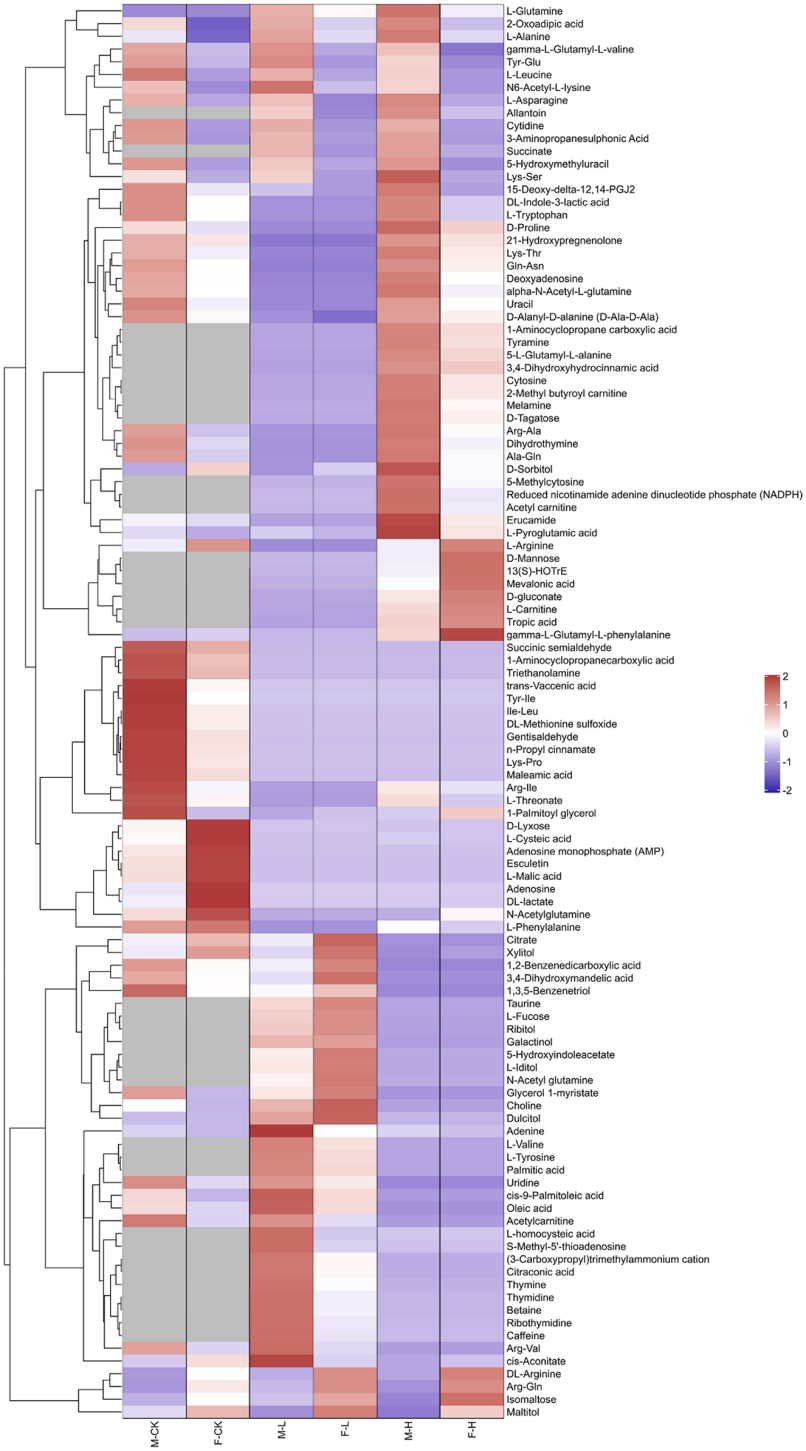
Clustering analysis of differential metabolites of male and female *Sargassum thunbergii* macroalgae under different UV-B radiation intensities. Rows and columns represent metabolites and treatment groups, respectively. The red color indicates a high abundance of a metabolite, whereas the blue color represents a low relative abundance of a metabolite. The abscissa represents samples and the ordinate represents differential metabolites and M-CK means: the control group of males; M-L means: males under low UV-B radiation conditions; M-H means: males under high UV-B radiation conditions; F-CK means: the control group of females; F-L means: females under low UV-B radiation conditions; and F-H means: females under high UV-B radiation conditions. Red means: males were higher than that of females. Blue means: females were higher than that of males.

To analyze the fundamental metabolic processes in macroalgae related to control, low, and high UV-B radiation and to determine the metabolic changes between females and males, we compared the metabolic profiles of females with those of males in the control group. In control group, there were 73 species metabolites contents have significant difference between male and female *S. thunbergii* comparison, which included that 54 metabolites content in males have significantly higher than females versus to 19 metabolites content in females have significantly higher than males. On the whole, amino acids, dipeptides, organic acids, and nucleic acids accounted for a large proportion (more than 72%), with 20, 14, 10, and 9 species, respectively (Figure 2A). For the male and female macroalgae under the low UV-B radiation treatment, 58 metabolites were detected, and there were 37 and 21 metabolites content have significantly higher in male and females, respectively. Amino acids, carbohydrates, organic acids, nucleic acids, and dipeptides accounted for a large proportion, with 11, 9, 8, and 8, respectively (Figure 2B). For male and female macroalgae under high UV-B radiation treatment, 65 metabolites have been demonstrated and there were 48 and 17 metabolites content have significantly higher in male and females, respectively. Amino acids, dipeptides, organic acids, and nucleic acids accounted for a large proportion, with 19, 12, 9, and 9, respectively (Figure 2C). There were 24 identical differential metabolites in the control group, low, and high UV-B radiation treatment (Figure 3), and most of the differential metabolites showed similar trends. These metabolites were mainly divided into two categories by hierarchical clustering analysis (Figure 4). Compared with the control, the first category was significantly upregulated under different UV-B radiation intensities, including most amino acids, nucleic acids, and lipids, while the content of the second category decreased with increasing UV-B intensity such as carbohydrates.

#### Pathways Associated With the Differentially Expressed Metabolites in Male and Female *Sargassum thunbergii* Macroalgae Under Enhanced UV-B Radiation

**Figure 5:**
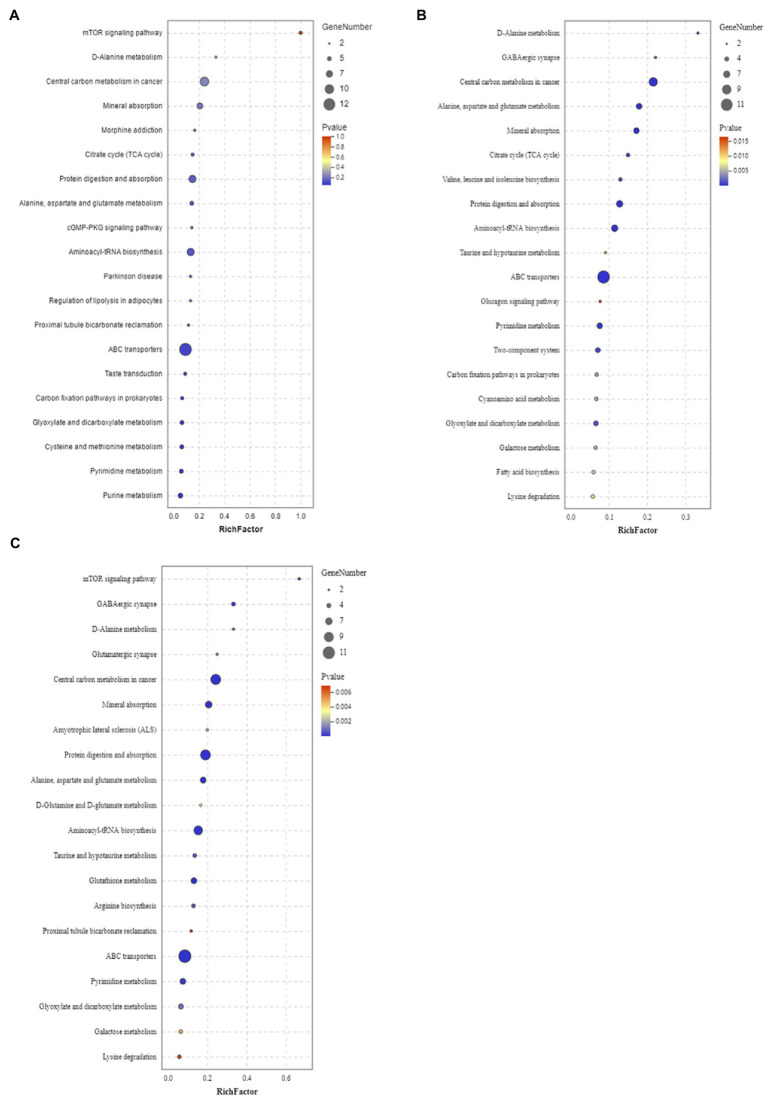
Enrichment analysis results of KEGG pathway in positive ion mode. Male and female *Sargassum thunbergii* in **(A)** control group; **(B)** low UV-B radiation conditions; and **(C)** high UV-B radiation conditions. The abscissa represents rich factors (calculated by the significantly regulated metabolites numbers of given metabolic pathway/all metabolite numbers of corresponding metabolic pathway) and the ordinate represents different metabolic pathways.

**Figure 6:**
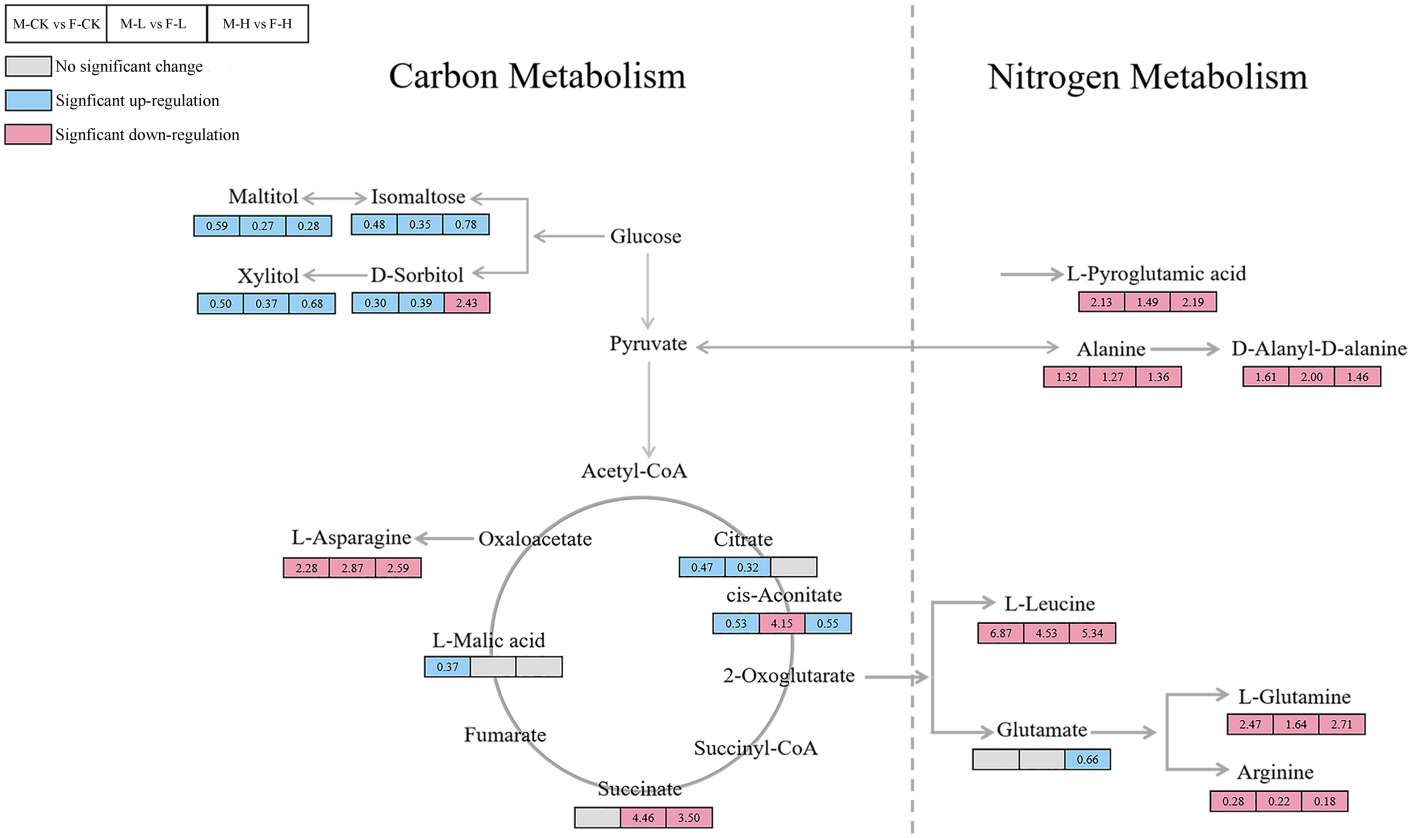
Summary of the significantly changed metabolites under control group, low, and high UV-B radiation. The values in the box represent the fold change of the metabolites; M-CK means: the control group of males; M-L means: males under low UV-B radiation conditions; M-H means: males under high UV-B radiation conditions; F-CK means: the control group of females; F-L means: females under low UV-B radiation conditions; and F-H means: females under high UV-B radiation conditions. Gray rectangle represents metabolite content has insignificant differences between male and female *Sargassum thunbergii*. Red rectangle represents metabolite content in males significantly higher than females. Blue rectangle represents metabolite content in females significantly higher than males.

The metabolic pathways that showed significant changes in females and males were amino acid metabolism, carbohydrate metabolism, and glutathione metabolism changed significantly after different UV-B radiation treatment. The relevant metabolic pathways are shown in Figure 5. The intermediates of the citric acid cycle, such as succinate, significantly increased under enhanced UV-B radiation, and cis-aconitate nd dsorbitol increased after an initial decrease. Levels of l-asparagine, l-glutamine, l-leucine, and d-alanine and most amino acids significantly decreased. Monosaccharides (such as mannose, d-tagatose, and d-lyxose), oligosaccharides (such as isomaltose and maltose), and sugar alcohols (such as maltitol, d-sorbitol, l-iditol, ribitol, and xylitol) were significantly downregulated under different UV-B radiation treatments. To more intuitively investigate the metabolic response of male and female *S. thunbergii* macroalgae to UV-B radiation, changes in target differential metabolites and related pathways were compared (Figure 6).

### Content Changes and Enzyme Activity of Important Substances

**Figure 7:**
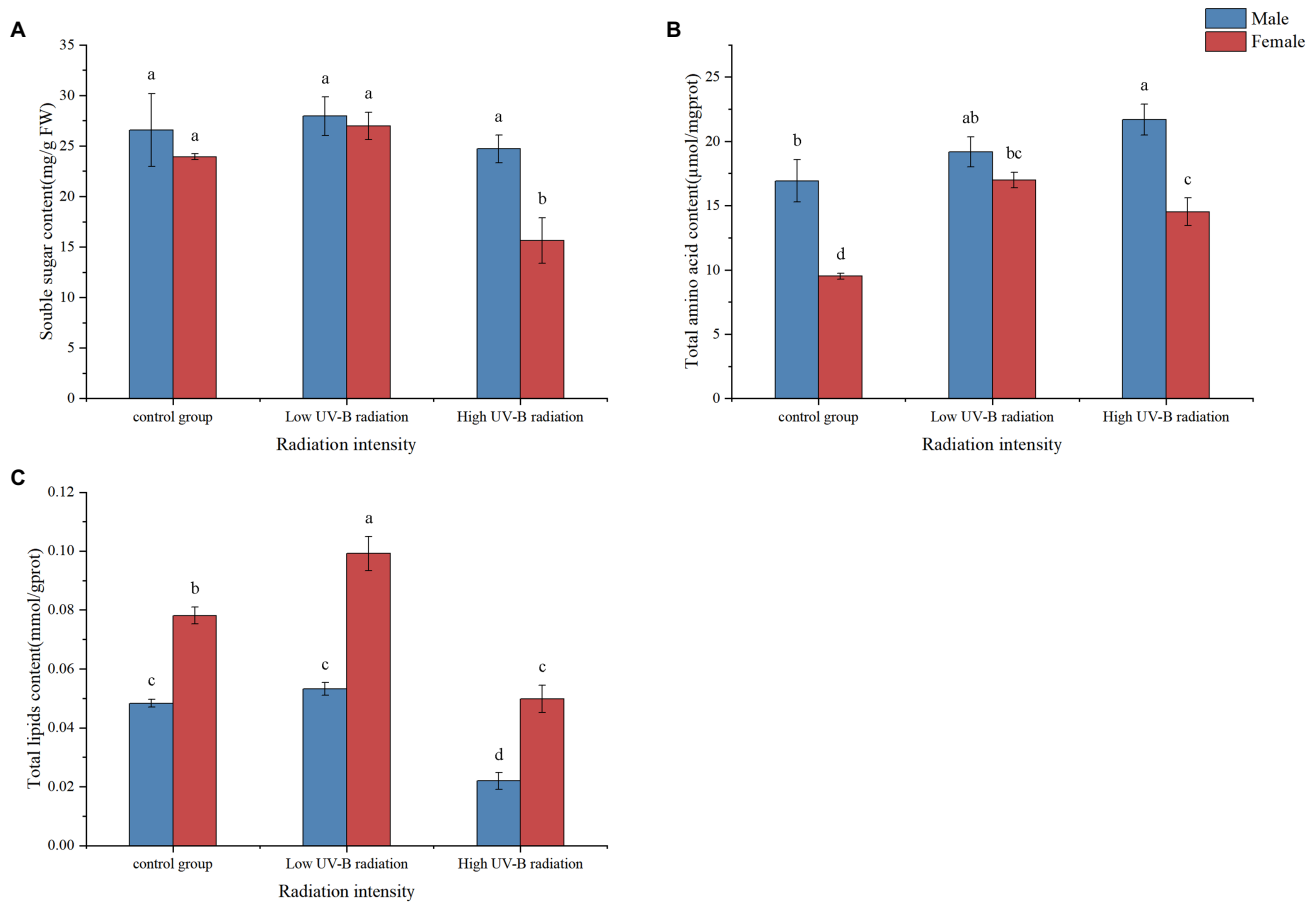
Soluble sugar **(A)**, total amino acid **(B)**, and lipid **(C)** content in male and female *Sargassum thunbergii* under control group and different intensities of UV-B radiation treatments. The values represent mean *n* ± SD (*n* = 3). Different letters mean significant differences between male and female *Sargassum thunbergii*.

Under low UV-B radiation, the total soluble sugar (TSS), total amino acid, and the lipid content of males *S. thunbergii* macroalgae have slightly higher than control group but not significantly (*p* > 0.05, Figure 7). The TSS content of female macroalgae under high UV-B radiation was lower than the control group but did not differ in male macroalgae. The total amino acid content of male and female macroalgae under high UV-B radiation was significantly higher than the control group. The lipid content of male and female macroalgae under high UV-B radiation was significantly lower than the control group.

**Figure 8:**
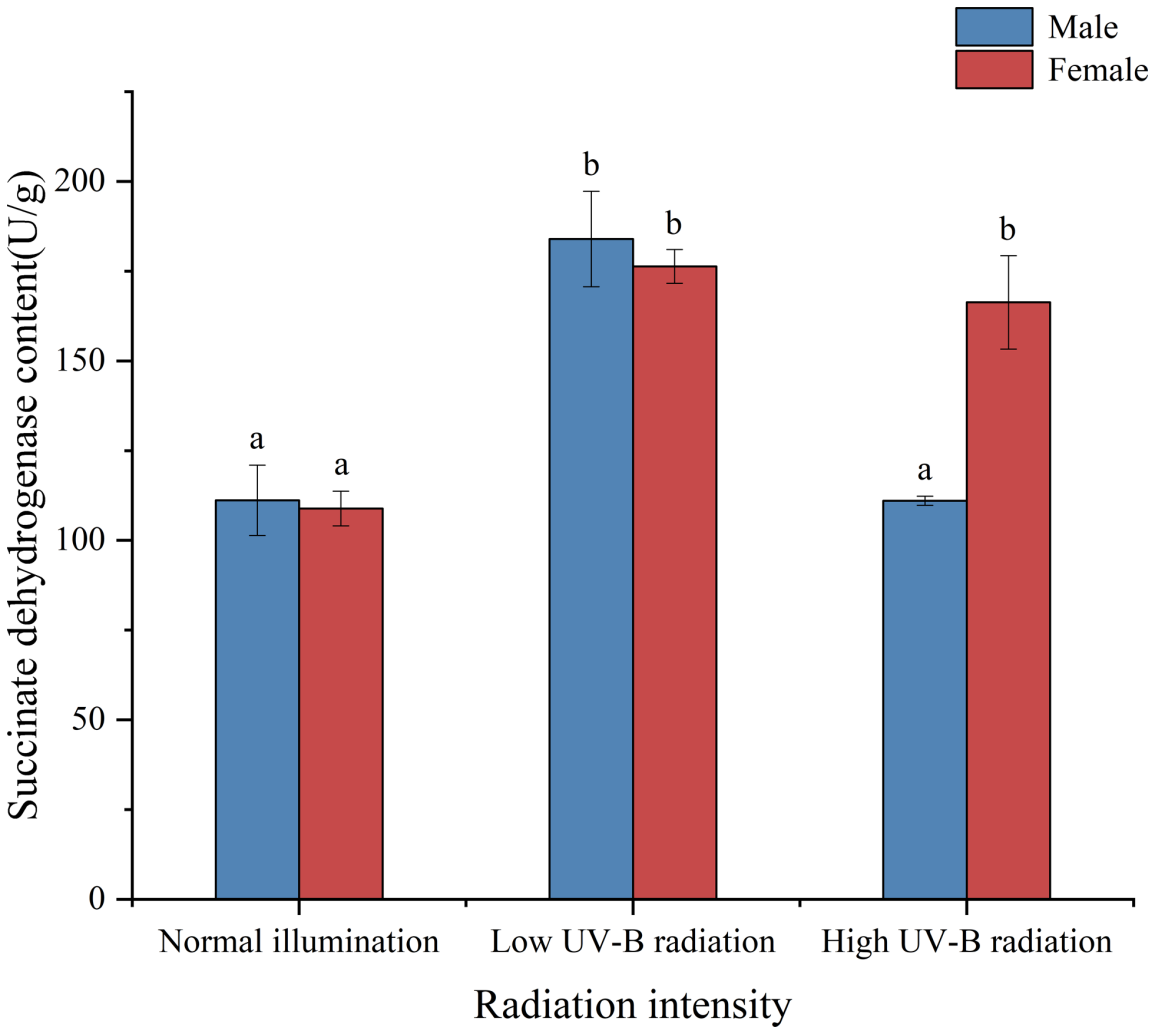
Effect of UV-B on the activities of succinate dehydrogenase in male and female *Sargassum thunbergii*. And the values represent mean *n* ± SD (*n* = 3). Different letters mean significant differences between male and female *Sargassum thunbergii*.

Succinate dehydrogenase is the only multi-subunit enzyme integrated on the membrane in the TCA cycle. In eukaryotes, it binds to the mitochondrial inner membrane and integrates into the cell membrane in prokaryotes. It is one of the hubs connecting oxidative phosphorylation and electron transfer. It provides electrons for the respiratory chain of oxygen demand and production of eukaryotic mitochondria and several protonuclear cells. It is also a marker enzyme of mitochondria. We found that succinate dehydrogenase activity (SDH) was induced in male and female *S. thunbergii* under low UV-B radiation. Until macroalgae exposing high UV-B radiation, there are significantly differences between male and females (*p* < 0.05, Figure 8). And the male SDH activities were recovered to the control group levels (*p* > 0.05, Figure 8).

## Discussion

Combining with metabolomics analysis, chlorophyll fluorescence parameters and carbon-based metabolites content measurement, the difference comparison in male and female *S. thunbergii* under enhanced UV-B radiation was processed and discussed. According to our results, it was found that males have more stable photosynthetic capacities and higher soluble sugar, amino acids content than females, which perform to closely intimate connection with the carbon and nitrogen metabolism and also provide us a direction for further experiments.

### Dioecious *Sargassum thunbergii* Chlorophyll Fluorescence Parameters Differences to the UV-B Radiation

Photosynthesis is the most basic physiological activity to photosynthetic organism, which can respond rapidly to the habitat changing (Hader et al., 2002). In our experiments, *F*_v_/*F*_m_ can directly reflect to the potential maximum photosynthetic capacity (Maxwell and Johnson, 2000), which could be decreased by adverse situations. Previous studies have reported that UV-B stresses not only lead to photoinhibition effects to photosynthesis, but also delay the photosynthetic recovery (Hanelt et al., 1997). Under UV-B radiation treatments, male and female *S. thunbergii* consistently have F_v_/F_m_ values decrease performances, especially for higher dose UV-B continuing 5 days. However, at the end of treatment, males still kept significantly higher F_v_/F_m_ values than females (9.28%, Supplementary Table S1), which meant that the photosynthetic capacity and photosynthesis stressed recovery abilities of males more stable than females under UV-B stresses (Beardall et al., 2001; Terada et al., 2016).

Additionally, Y(NPQ) and Y(NO) values reflect to the energy dissipation abilities of PSII systems (Kramer et al., 2004) and upregulated Y(NPQ) generally means that photoautotroph is suffered to stressed conditions and they could activate to their defend mechanisms (Peter and Rolf, 2005; Barták et al., 2018). However, when the stressed influences exceed to the tolerance limits, macroalgal Y(NPQ) could not increasing and their energy dissipation abilities will lose efficacy as well as Y(NO) will increase significantly (Kramer et al., 2004). Under high UV-B treatments and 5 day treatments, Y(NPQ) of males shown significant higher than females, which consistent with the *F*_v_/*F*_m_ results. With the UV-B dose increasing and treatments time extension, Y(NO) of male and female algae also expressed significant differences. These results indicated that the UV-B radiation suppressed females PSII light conversion abilities more obvious than males versus to the light damage more severe than males.

### Metabonomics Analysis

Metabolomics are an essential part of system biology, by analyzing the changes of endogenous small molecule metabolites caused by environmental stimuli; the relationship between physiological function and metabolism can be investigated. With the continuous improvement of detection and identification techniques for metabolites, metabolic regulation in response to abiotic stress has attracted widespread attention (Pineau et al., 2013; Zhao et al., 2015). In the past decade, new progress has been made by metabolomic techniques, but these mainly concentrated on land plants and commercial crops (Fernie and Schauer, 2009; Saito and Matsuda, 2010; Dong et al., 2015; Omranian et al., 2015). As a commonly environmental stress, UV-B radiation does apparent damage to intertidal species and functioned to the materials of protein, DNA, and photosynthetic pigments in photoautotroph (Arts and Rai, 1997). For past studies, there were seldom studies reported to compare different regulation mechanisms in dioecious macroalgae. So we mainly analyzed the differences in the response of male and female macroalgae to enhanced UV-B radiation from the perspective of metabolomics.

In regulated processes, there were a series of evidence proved that direct and indirect effects of UV-B radiation adversely affect the primary metabolism, including reduction in photosynthesis and carbohydrate production, increase in organic and amino acids, and disruption of pigments and proteins (Pego et al., 2000; Day and Neale, 2002; Kusano et al., 2011). Accordingly, the metabolomic analysis can capture global changes responding the differences between males and females. Based on our results, specific metabolites involved in tricarboxylic acid cycle, carbohydrate metabolism, and amino acid metabolism have been detected which also were member in carbon and nitrogen metabolism (Figures 5, 6). Under high UV-B radiation stimulation, males have significant higher soluble sugar and amino acid content than females (Figure 7), which biosynthesized from the primary carbon and nitrogen metabolism. And the biochemical measurement results also pointed to that the fundamental C and N metabolism served as the obvious pathways responding to UV-B radiation and their corresponding metabolites accumulated regulation also shown gender differences.

### Carbon Metabolism Responding to Enhanced UV-B Radiation

As an important energy substance in plants, sugar is the material source for growth and development, and also the energy basis for cell growth. Carbohydrate metabolism is quickly modulated in response to environmental changes. As an important energy substance, carbohydrate metabolism is closely related to the response of plant physiological activities to the environment. For example, carbohydrates can directly provide energy for stress response, and amino acids can respond to environmental stress as precursors of related metabolites (Rolland et al., 2006; Martínez et al., 2014; Hildebrandt et al., 2015). Therefore, changes in organisms, soluble sugar content can reflect the sum of the impact of environment on energy metabolism and the level of individual adaptability under stress to a certain extent. Additionally, succinic dehydrogenase (SDH) is a critical enzyme that determines the flow direction of the carbon-skeletons in the TCA cycle, and it is the only enzyme embedded in the mitochondrial inner membrane. We found that with the increase of radiation, the SDH activity in males and females increased at first and then decreased. Previous studies showed that the TCA cycle as the trunk pathways for respiratory metabolism and the pentose phosphate pathway as the branch pathway for respiratory metabolism (Lin et al., 2016, 2018; Zhang et al., 2017a). Plants generate energy by increasing respiration to produce defensive compounds to cope with oxidative stress. With the increase of radiation, the TCA cycle marker enzyme (SDH) activity decreased, suggesting that the TCA acid cycle was blocked and energy metabolism decreased. The SDH activity was lower in males than in females under the high UV-B radiation and showed the consistent with the results of metabolomics. The succinic acid in male macroalgae is upregulated compared with female macroalgae under UV-B radiation. Metabolic map analysis of macroalgae shows that the TCA cycle of female macroalgae is slower than male at enhanced UV-B, and the level of organic acids involved in the TCA cycle decreases. Glycolysis in cytoplasm and the TCA cycle in mitochondria are major sources for ATP (Chen et al., 2020). Under high UV-B radiation conditions, ATP is required for many biological processes, including ion transport, ROS scavenging, and UV-absorbing compound synthesis (Zhang et al., 2017b). Combining with the soluble sugar content measurement, under high UV-B radiation, males content was significantly higher than females, which might attributed to their preferable photosynthetic abilities. The higher PAM parameters mean the more effective photosynthesis in males and more quickly carbohydrate accumulated rates than females under UV-B radiation. Meanwhile, soluble sugars as the common stressed resistance materials could directly transferred in cells keeping stable osmotic pressure as well as maintained other metabolisms proceeding. And soluble sugars also served as energy storage materials rapidly assimilated and supported normal intracellular physiological activities. From the information given in the above, more quantities soluble sugar indicated that males possessed more stable regulated systems copying with stressed conditions and also ensured more abundant energy supply to activate defense mechanisms and self-repairing functions.

In this study, the contents of lyxose, xylose, d-sorbitol, maltitol, and isomaltose in male macroalgae were significantly lower than that in female macroalgae, respectively. These sugars are also important products of carbon metabolic pathways. The efficient role of sugars as true ROS scavengers during abiotic stress has been proved. The synergistic interaction of sugars functions as an integrated redox system in plants, scavenging ROS, enhancing stress tolerance (Van den Ende and Valluru, 2008; Furlan et al., 2012). It is believed that the catabolism of glucose also provides energy for plant growth and reduces oxidative damage (Mortimer et al., 2011). Therefore, we speculated that the reason for the downregulation of carbohydrates in male plants compared with that in female plants was that the carbohydrates consumed by male plants for scavenging ROS were more than those consumed by female plants, so the accumulation amount was lower than that of female plants, indicating downregulation. Under low UV-B radiation, the soluble sugar content trend measured of male and female macroalgae showed the same trend, but under high UV-B radiation, the soluble sugar content measured of male macroalgae increased significantly compared to female plants.

The quantity and quality of lipid and their entirely biochemical composition vary in response to environmental conditions (Liu et al., 2008; Chen et al., 2011). Under unfavorable environmental or stressed conditions, macroalgae regulated to their lipid biosynthetic pathways toward the formation of neutral lipids (Hu et al., 2008; Lörling et al., 2010), in which mainly accumulate as the triglyceride (TAG, Alboresi et al., 2016; Légeret et al., 2016) responding to adverse environment. Though in control group, females also have significantly higher performance than males and nothing has changed in low and high UV-B treatments. Combing with the lower PAM parameters Y(II) (true photosynthesis rates), females kept allocating more energy flowing into fatty acid carbon chains elongation under UV-B radiation as well as for energy storing than males. Comparing to soluble sugars, lipid served as the stable metabolites for energy storing and their decomposing also needed more enzymes, and slower than single chain carbohydrates. Obviously, under UV-B radiation, females flowed into less energy and spend more for lipid biosynthesis than males. In higher plants, this regulation similarly confirmed by the spermatophyte species (Korpelainen, 1992) and they usually allocate more energy to generative organs prior to its productive stage and show lower tolerance abilities to stressed conditions (Xu et al., 2008). In our experiments, lipid content regulation different from soluble sugar and amino acid have been proved in brown macroalgae responding to UV-B perturbation.

### Nitrogen Metabolism Responding to Enhanced UV-B Radiation

Nitrogen metabolism and carbon metabolism are closely related. The synthesis of nitrogen metabolites needs carbon skeleton, and the process of carbon metabolism needs the promotion of nitrogen metabolites. The relationship between carbon and nitrogen metabolism is dynamic, and both promote and restrict each other. For example, nitrogen absorption, metabolism, and transformation require photosynthesis to provide energy, and the smooth progress of photosynthesis and carbon fixation also requires nitrogen metabolism to provide key enzymes and proteins. Macroalgal carbon and nitrogen metabolism network and its interaction directly affect the growth, development, and the defense function to environmental stress. Nitrogen metabolism is the main pathway of amino acid and protein synthesis, amino acid is not only an important substrate for protein synthesis, but also a precursor for stress response. Research has shown that UV-B radiation promotes the synthesis of proteins and the metabolism of pigments. Proline is an effective ROS scavenger, proving that proline plays a vital role in the redox of algal cells under stress conditions (Ashraf and Foolad, 2007). As a branched-chain amino acid, valine can scavenge the generated reactive oxygen species (ROS; Hu et al., 2015). In addition, it is directly and indirectly involved in many important metabolic functions, including aminoacyl-tRNAs biosynthesis, which is part of the protein synthesis mechanism (Ling et al., 2009; Han et al., 2019). Aromatic amino acids are precursors for the synthesis of flavonoids, and their enhanced synthesis will increase the protein content (Kreft et al., 2002). In addition, amino acids can function as the precursors of secondary metabolites that can protect plants from various stresses (Häusler et al., 2014).

Amino acids play an essential role in regulating plant physiological processes, such as acting as osmotic agents, regulating ion transport, regulating stomatal opening, and acting as precursors and signal transduction substances to defense the synthesis of related metabolites. When encountering stress, plants usually control the absorption, synthesis, and degradation of amino acids to reduce the harm caused by stress (Florencio et al., 2018). Most of the amino acids in male and female macroalgae were upregulated under UV-B radiation, especially l-valine, d-proline, l-phenylalanine, l-glutamine, l-alanine, and l-leucine. The results of our physiological experiments show that the total amino acid content of males was higher than females under the high or low UV-B radiation, and it is consistent with the results of metabolomic analysis.

In our metabolomic results, under UV-B radiation males, aspartic acid and alanine content have significantly higher than females, which might have closely connections to their defense mechanisms and also reflected in specific amino acids species synthesizing. Alanine and aspartic acid are derivatives of glycolysis and TCA cycle, respectively. The significant increase in intracellular alanine and aspartate content reflects the positive response of these energy metabolism pathways (Kim et al., 2018), which also shown that the males attached more resilient to UV-B radiation than females. It was also found that the tissue nitrogen content of brown macroalgae under UV radiation was actually higher than normal conditions and their amino acid content also has increased (Pavia et al., 1997). For keeping normally physiological activities, nitrogen metabolism acted an indispensable role in dioecious *S. thunbergii*, as the nitrogen-based metabolites, amino acids not only functioned in stress resistance but also shown significantly gender difference in their characteristic biosynthesis and content accumulation.

## Conclusion

Male and female *S. thunbergii* have shown different responds in photosynthetic physiological characteristics and metabolites regulation after UV-B radiation exposure. Males have significantly superiority performance in the chlorophyll fluorescence parameters of *F*_v_/*F*_m_, Y(II), and Y(NO) either low or high UV-B radiation treatments. Based on metabonomics analysis, the main types of metabolites are amino acids, sugar nucleotides, and organic acids, which were the significant intermediates of macroalgae’s principally metabolic processes, including glycolysis, TCA cycle, and amino acid biosynthesis pathways. Exposing UV-B radiation conditions, the amino acid and energy metabolism of male macroalgae were enhanced compared with female macroalgae, which might help the males behaved better ability to resist and defend the UV-B stresses. And the metabolomics analysis also presented that *S. thunbergii*’s carbon and nitrogen metabolic pathways were the obvious responding regulation to UV-B stresses. The results of our experimental measurement also showed that under high UV-B radiation, there were significant differences in soluble sugar, total amino acid, lipid contents, and SDH enzymic activities between male and female macroalgae. Corresponding to the metabonomic analysis, the physiological and biochemical measurements, we could conclude that male *S. thunbergii* depended on their specifically metabolic processes, such as photoprotective mechanisms, intracellular osmotic pressure regulations, and stressed resistance metabolites accumulation to behave more stable abilities than females responding to UV-B perturbations. Compared with the previous studies (usually regardless of macroalgal genders), our studies will realistically and effectively reflect to brown macroalgae adopting mechanisms in defending UV-B stress, and also help us rediscovered the sexual differences in dioecious macroalgae.

## Data Availability Statement

The original contributions presented in the study are included in the article/**Supplementary Material**, further inquiries can be directed to the corresponding authors.

## Author Contributions

YS, QL, SS, and PL: conceptualization. YS, QL, SS, YZ, and XT: methodology. YS: writing—original draft preparation and data curation. QL, JC, SS, and PL: resources and investigation. QL: visualization. JC, SS, and PL: formal analysis. YZ and XT: validation, supervision, funding acquisition, writing—review and editing, and project administration. All authors contributed to the article and approved the submitted version.

## Funding

This research was funded by the NSFC-Shandong Joint Fund (U1806213), the National Key R&D Program of China (2019YFD0901204), and the National Natural Science Foundation of China (42176154).

## Conflict of Interest

The authors declare that the research was conducted in the absence of any commercial or financial relationships that could be construed as a potential conflict of interest.

## Publisher’s Note

All claims expressed in this article are solely those of the authors and do not necessarily represent those of their affiliated organizations, or those of the publisher, the editors and the reviewers. Any product that may be evaluated in this article, or claim that may be made by its manufacturer, is not guaranteed or endorsed by the publisher.

## Footnotes

^1^ http://www.kegg.jp/

^2^ http://cloud.aptbiotech.com/
