# Supplementary material for: Physiological Responses and Metabonomics Analysis of Male and Female Sargassum thunbergii Macroalgae Exposed to Ultraviolet-B Stress
Source: Front Plant Sci. 2022 Apr 4;13:778602. doi: 10.3389/fpls.2022.778602 (PMC9037290; doi:10.3389/fpls.2022.778602)
Supplement: Supplementary file 1 [file Table_1.DOCX]

Table S1 PS II fluorescence parameters of male and female *S. thumbergii* macroalgae under different UV-B radiation intensities

|  |  | Fv/Fm | | | Y(Ⅱ) | | | Y(NPQ) | | | Y(NO) | | |
| --- | --- | --- | --- | --- | --- | --- | --- | --- | --- | --- | --- | --- | --- |
|  |  | male | female | male than  female  (*p* values) | male | female | male than  female  (*p* values) | male | female | male than  female  (*p* values) | male | female | male than  female  (*p* values) |
|  | Control group | 0.6618  ±0.0025 | 0.6670  ±0.0051 | -0.78%  (0.067) | 0.4993  ±0.0035 | 0.4803  ±0.0061 | 3.97%^*^  (0.016) | 0.2215  ±0.0030 | 0.2221  ±0.0028 | -0.26%  (0.883) | 0.3580  ±0.0030 | 0.3646  ±0.0055 | -1.81%  (0.182) |
| Day 0 | Low UV-B | 0.6601  ±0.0035 | 0.6682  ±0.0020 | -1.03%  (0.127) | 0.4963  ±0.0020 | 0.4776  ±0.0037 | 3.93%^*^  (0.012) | 0.2272  ±0.0045 | 0.2232  ±0.0056 | 1.79%  (0.394) | 0.3521  ±0.0065 | 0.3662  ±0.0050 | -3.86%  (0.122) |
|  | High UV-B | 0.6612  ±0.0056 | 0.6684  ±0.0047 | -3.36%  (0.06) | 0.4986  ±0.0037 | 0.4765  ±0.0037 | 4.63%^*^  (0.002) | 0.2246  ±0.0030 | 0.2214  ±0.0035 | 1.44%  (0.283) | 0.3562  ±0.0070 | 0.3651  ±0.0065 | -2.50%  (0.123) |
|  | Control group | 0.6597  ±0.0051 | 0.6683  ±0.0051 | -1.30%  (0.056) | 0.4807  ±0.0047 | 0.4717  ±0.0058 | 1.91%  (0.107) | 0.2207  ±0.0035 | 0.2176  ±0.0055 | 1.38%  (0.471) | 0.3567  ±0.0043 | 0.3657  ±0.0045 | -2.64%  (0.082) |
| Day 1 | Low UV-B | 0.6650  ±0.0030 | 0.6623  ±0.0041 | 0.40%  (0.478) | 0.4973  ±0.0035 | 0.4697  ±0.007 | 5.89%^*^  (0.001) | 0.1933  ±0.0035 | 0.2087  ±0.0043 | -7.50%^*^  (0.005) | 0.3663  ±0.0050 | 0.3707  ±0.0015 | -1.17%  (0.227) |
|  | High UV-B | 0.5897  ±0.0040 | 0.5697  ±0.0057 | 3.51%  (0.049) ^*^ | 0.4057  ±0.0041 | 0.3977  ±0.0041 | 2.01%  (0.078) | 0.1722  ±0.0043 | 0.1648  ±0.0040 | 4.24%  (0.110) | 0.4377  ±0.0061 | 0.4451  ±0.0072 | -1.65%  (0.226) |
|  | Control group | 0.6527  ±0.0047 | 0.6529  ±0.0032 | -0.04%  (0.947) | 0.4683  ±0.0030 | 0.4292  ±0.0020 | 9.09%^*^  (0.001) | 0.1983  ±0.0055 | 0.1894  ±0.0026 | 4.94%  (0.057) | 0.3747  ±0.0030 | 0.3753  ±0.0020 | -0.09%  (0.882) |
| Day 3 | Low UV-B | 0.6017  ±0.0085 | 0.5813  ±0.0043 | 3.51%^*^ (0.027) | 0.4258  ±0.0043 | 0.3966  ±0.0060 | 7.30%^*^  (0.003) | 0.1713  ±0.0062 | 0.1645  ±0.0020 | 4.27%  (0.138) | 0.4007  ±0.0055 | 0.4474  ±0.0020 | 10.36%^*^  (0.002) |
|  | High UV-B | 0.5207  ±0.0075 | 0.4751  ±0.0057 | 9.58%^*^  (0.001) | 0.3843  ±0.0047 | 0.3633  ±0.0056 | 5.78%^*^  (0.008) | 0.1519  ±0.0050 | 0.1488  ±0.0036 | 2.62%  (0.363) | 0.4513  ±0.0062 | 0.5023  ±0.0011 | 10.22%^*^  (0.015) |
|  | Control group | 0.6327  ±0.0040 | 0.6449  ±0.0040 | -1.90%  (0.122) | 0.4316  ±0.0067 | 0.4113  ±0.0135 | 4.95%^*^  (0.039) | 0.1617  ±0.0050 | 0.1543  ±0.0030 | 4.98%  (0.086) | 0.3963  ±0.0025 | 0.3787  ±0.0085 | 4.66%  (0.053) |
| Day 5 | Low UV-B | 0.5783  ±0.0050 | 0.5473  ±0.0055 | 5.66%^*^  (0.002) | 0.4076  ±0.0023 | 0.3675  ±0.0075 | 10.91%^*^  (0.002) | 0.1529  ±0.0050 | 0.1433  ±0.0077 | 6.66%  (0.079) | 0.4317  ±0.0030 | 0.4842  ±0.0062 | 10.81%^*^  (0.005) |
|  | High UV-B | 0.4773  ±0.0060 | 0.4368  ±0.0051 | 9.28%^*^  (0.001) | 0.3589  ±0.0045 | 0.3291  ±0.0045 | 9.05%^*^  (0.005) | 0.1367  ±0.0040 | 0.1243  ±0.0056 | 9.93%^*^  (0.03) | 0.4753  ±0.0032 | 0.5412  ±0.0040 | 12.17%^*^  (0.005) |

Significant differences (*p* < 0.05) between males and females are indicated by an asterisk (*). [Negative](javascript:;) [value](javascript:;) means female values higher than males.
